# The interaction of HspA1A with TLR2 and TLR4 in the response of neutrophils induced by ovarian cancer cells in vitro

**DOI:** 10.1007/s12192-012-0338-2

**Published:** 2012-04-24

**Authors:** Magdalena Klink, Marek Nowak, Michał Kielbik, Katarzyna Bednarska, Edyta Blus, Marian Szpakowski, Krzysztof Szyllo, Zofia Sulowska

**Affiliations:** 1 Institute of Medical Biology, Polish Academy of Sciences, Lodowa 106, 93-232 Lodz, Poland; 2 Department of Gynecology, Polish Mother’s Memorial Hospital—Research Institute, Lodz, Poland; 3 Department of Operative Gynecology, Polish Mother’s Memorial Hospital—Research Institute, Lodz, Poland

**Keywords:** Heat shock protein, Toll-like receptors, Neutrophils, Ovarian cancer cells

## Abstract

Inducible heat shock protein (HspA1A) promotes tumor cell growth and survival. It also interacts with effector cells of the innate immune system and affects their activity. Recently, we showed that the direct contact of ovarian cancer cells, isolated from tumor specimens, with neutrophils intensified their biological functions. Our current experiments demonstrate that the activation of neutrophils, followed by an increased production of reactive oxygen species, by cancer cells involves the interaction of HspA1A from cancer cells with Toll-like receptors 2 and 4 expressed on the neutrophils’ surface. Our data may have a practical implication for targeted anticancer therapies based, among other factors, on the inhibition of HspA1A expression in the cancer cells.

## Introduction

Mammalian heat shock proteins (Hsp) have been classified into five families, according to their molecular size. The Hsp70 family includes, among others, heat inducible Hsp70i (HspA1A), formerly known as Hsp72, and constitutively expressed Hsc70 (HspA8) proteins (Kampinga et al. 2009). Intracellularly located HspA1A mainly participates in the maintenance of cellular homeostasis during stress conditions such as heat, hypoxia, ischemia, acidosis, and energy depletion. It is also known that HspA1A is secreted from dead and living cells and exerts a powerful effect on the immune system (Kregel 2002; Daugaard et al. 2007). HspA1A is released from necrotic cells via a passive mechanism or from living cells via an active mechanism involving the translocation of protein through the lysosomal compartment, using the ABC family of transmembrane transporters (Mambula and Calderwood 2006; Asea 2007a; Mambula et al. 2007). The released HspA1A, by being bound with the various immune cells, participates in the inflammatory and immune responses. It also induces the production of pro-inflammatory cytokines, binds the antigenic peptides, and stimulates the adaptive immune response, as well as enhances the expression of MHC class II on dendritic cells, which in turn allows its maturation (Asea et al. 2002; Calderwood et al. 2007a, 2005). HspA1A interacts with target cells via a number of cell surface receptors, e.g., CD91, CD36, CD40, and Toll-like receptors (TLRs) 2 and 4 or other c-type lectin receptors (Calderwood et al. 2007b; Theriault et al. 2005).

TLR2 and TLR4 are type 1 transmembrane proteins that belong to the family of pattern recognition receptors. The signaling pathway induced via TLRs is a multistep process that may or may not depend on MyD88 adapter protein pathways. An adapter protein, MyD88, binds to a receptor Toll/IL-lR-domain and interacts with protein kinases of the IRAK family (e.g., interleukin-1 receptor-associated kinases—IRAK1, IRAK4), which leads to the phosphorylation of the target signaling proteins, including mitogen-activated protein kinases, phosphoinositide 3-kinase, nuclear factor-κB (NF4-κB), and AKT (also known as protein kinase B) (Chen et al. 2007; Krishnan et al. 2007).

Numerous reports demonstrate that basal levels of HspA1A, both in a serum and in a tumor microenvironment of patients with various cancers (breast, lung, prostate, colorectal, pancreatic), often correlate with poor survival and response to chemotherapy of patients (Abe et al. 2004; Suzuki et al. 2006; Zhang et al. 2009; Sherman and Multhoff 2007; Aghdassi et al. 2007). HspA1A located intracellularly seems to be necessary for cancer cell growth and survival. It protects cancer cells from oxidative stress, hypoxia, and inflammatory cytokines and blocks the apoptotic pathways in cells (Garrido et al. 2006; Daugaard et al. 2005). On the other hand, HspA1A is highly expressed on the cell surface of tumor cells, but not on normal cells, as an integral membrane-bound protein that can be uniquely identified by cmHsp70.1 monoclonal antibody (Sherman and Multhoff 2007; Multhoff and Hightower 2011). HspA1A is also secreted into extracellular space (Kleinjung et al. 2003; Schmitt et al. 2007; Asea 2007b). HspA1A located extracellularly, as well as when membrane-bound, can trigger the activity of immune cells and initiate an immune response. It was found that cancer cells demonstrating membrane-bound HspA1A stimulated the migration and cytolytic activities of natural killer cells (Asea 2007b; Botzler et al. 1996; Gastpar et al. 2005; Multhoff and Hightower 1996). Among the immune cells that interact with cancer cells are polymorphonuclear cells (neutrophils). As others have shown, cytotoxic mediators, such as reactive oxygen species (ROS), released from neutrophils, could not only mediate the tumor cells lysis (Di Carlo et al. 2001; Lichtenstein et al. 1989; Lopez-Lazaro 2007) but could also promote tumor progression (De Larco et al. 2004).

We found previously that the ovarian cancer cells, isolated from the tumors of patients with advanced stage ovarian cancer, enhanced the production of ROS by autologous peripheral blood neutrophils (Klink et al. 2008). However, the molecules, receptors, etc. which mediate this interaction have not yet been pointed out. The aim of this study was to examine whether HspA1A membrane-bound and/or released from ovarian cancer cells could be involved in the activation of neutrophils of patients with advanced ovarian cancer. The participation of TLR2 and TLR4 expressed on neutrophils in the interaction via HspA1A with ovarian cancer cells was also investigated.

## Materials and methods

### Chemical reagents and antibodies

Histopaque 1077, Percoll, phorbol 12-myristate 13-acetate (PMA), formyl-methionyl-leucyl-phenylalanine (fMLP), bovine serum albumin (BSA), propidium iodide (PI), dimethyl sulfoxide (DMSO), Triton X100, mouse IgG2a anti-β actin, horseradish peroxidase (HRP)-conjugated goat anti-mouse IgG, IgA, IgM, NaF, EGTA, EDTA, NaCL, phenylmethylsulfonyl fluoride (PMSF), sodium orthovanadate, protease inhibitor cocktail, Tris, sodium dodecyl sulfate (SDS), β-mercaptoethanol, glycerol, glycine, bromophenyl blue, methanol, polyethylene glycol 3500 (PEG 3500), Tween 20, and IRAK1/4 inhibitor were purchased from Sigma-Aldrich (USA). Luminol was obtained from Serva (Germany). DC Protein Assay Kit, 30 % acrylamide/Bis-37.5:1, polyvinylidene difluoride (PVDF) membrane, and Precision Plus WesternC standard were purchased from BioRad (USA). Dextran T500 was obtained from Pharmacosmos A/S (Denmark). Purified human recombinant Hsp72 (hrHsp72) was purchased from Stressgen (USA). Phosphate-buffered saline (PBS) was obtained from BIOMED-Lublin (Poland). Hanks’ balanced salt solution (HBSS), RPMI 1640 supplemented with 2 mM of glutamine, and 0.25 % Trypsin solution with EDTA (Trypsin–EDTA solution) were purchased from PAA, the Cell Culture Company (Austria). Antibodies mouse IgG2a anti-human TLR2 sodium azide free, mouse IgG2a anti-human TLR4 sodium azide free, mouse IgG2a anti-TLR2 PE-conjugated, and mouse IgG2a anti-TLR4 FITC conjugated were obtained from Imgenex (USA). Mouse IgG1 anti-human Hsp72 (SPA810) and mouse anti-human Hsp72 (SPA810) FITC were purchased from Stressgen (USA). Antibody to Epithelial Cell Adhesion Molecule (EpCAM) FITC-conjugated was purchased from Miltenyi Biotec GmbH (Germany). Mouse IgG1 monoclonal antibody cmHsp70.1 FITC conjugated was obtained from Multimmune, (Germany). Hsp70 ELISA Kit was purchased from Stressgen (USA). BD CellFIX solution was obtained from BD Biosciences Pharmingen (USA).

### Patients

This study included patients suffering from advanced epithelial ovarian cancer (International Federation of Gynecology and Obstetrics (FIGO) III/IV). The patients had undergone the primary surgery for ovarian cancer in the Gynecologic Surgery Department and Operative Gynecology Department of the Polish Mother’s Memorial Hospital—Research Institute, Lodz, Poland. The patients’ ages ranged from 35 to 70 years, and the ovary was a primary site of malignancy for all the women. All tissue samples removed during the surgery were examined by a pathologist, and the tumor grade and its histological type were established according to the FIGO and WHO classification (Benedet et al. 2000). None of the patients received any chemo- or radiotherapy before the surgery. The control group consisted of 10 age-matched healthy female volunteers.

### Cell lines description

Ovarian cancer cell lines of epithelial morphology and adherent growth type were used. SK-OV-3 and OVCAR-3 cell lines were purchased from the American Type Culture Collection (Manassas, VA, USA). A2780 cell line was obtained from the European Collection of Cell Cultures (Porton Down, Salisbury, UK). Cells were cultured in a culture medium (CM) containing RPMI 1640 medium supplemented with 2 mM glutamine and 10 % fetal bovine serum at 37 °C and in a 5 % CO_2_ atmosphere. CM renewal was every 2 to 3 days, and cells were passaged two to three times a week, trypsinized using Trypsin–EDTA solution at 37 °C and in a 5 % CO_2_ atmosphere.

### Isolation of neutrophils

Peripheral venous blood from cancer patients was taken on the day of surgery, before the surgical procedure. Blood samples from the cancer patients and the control group were centrifuged on Histopaque 1077 (400×*g*, 30 min, room temperature). Mononuclear cells were collected and saved. Remaining plasma and gradient material were aspirated down to the erythrocytes interface. Cell pellets, containing erythrocytes and neutrophils, were resuspended in PBS to the initial whole blood volume. Next, red blood cells were allowed to sediment for 30 min at room temperature by the addition of equal amounts of 3 % Dextran T500. The neutrophil-rich supernatant was centrifuged (500×*g*, 10 min), and neutrophils’ pellet was washed twice with PBS without Ca^+2^ and Mg^+2^ ions. The remaining erythrocytes that contaminate the neutrophils’ pellet were lysed with NH_4_Cl lysing solution. Finally, cells were resuspended in HBSS buffer. Neutrophil viability (>95 %) and cell purity (>98 %) were assessed by Trypan blue exclusion and May–Grünwald–Giemsa staining, respectively.

### Isolation of ovarian cancer cells

Ovarian cancer tissue was collected during the surgery from the primary site. Ovarian cancer (OC) cells were obtained as described previously (Klink et al. 2008). Briefly, fragments of tumor specimens were rubbed by using the Cell Dissociation Sieve—Tissue Grinder Kit (Sigma) directly to PBS. Then, cells were washed three times with PBS (300×*g*, 10 min, 4 °C) and were filtered using Filcon disposable filter device (BD Biosciences, USA). Finally, cells were layered on a discontinuous Percoll gradient (60, 50, 40, and 30 %) and then centrifuged (500×*g*, 30 min, room temperature) to remove the contaminating leukocytes and red blood cells. Interface 0 (between the PBS and 30 % Percoll) contained >95 % pure tumor cells. The OC cells were washed twice with PBS and resuspended in HBSS. The cytocentrifugal preparations of OC cells were prepared, and the purity of cells was assessed by a hematoxylin–eosin histological and a leukocyte common antigen staining. To confirm the epithelial origin of cancer cells and for their separation from other cell types, cells were labeled with anti-EpCAM FITC antibody. EpCAM expression on tumor cells was detected by flow cytometry method (FCM). OC cell viability was determined using a PI exclusion assay.

### Flow cytometry

Surface expression of TLR2, TLR4, and membrane-bound HspA1A was assessed by flow cytometry. Freshly isolated neutrophils (2 × 10^6^ cells/ml in HBSS) were incubated with 20 μg/ml of anti-TLR2 and anti-TLR4, sodium azide-free, blocking monoclonal antibodies (mAbs) for 30 min, at room temperature or remained untreated. Then, cells were stimulated or not with 1 μg/ml of PMA or 1 μM of fMLP for 30 min at 37 °C in a 5 % CO_2_ atmosphere. Placing all samples on ice stopped the reaction and cells were then stained with anti-TLR2 PE-conjugated and anti-TLR4 FITC-conjugated mAbs or with 10 μl of appropriate isotype control for 30 min at 4 °C in the dark.

OC cells were stained with cmHsp70.1 FITC-conjugated mAbs that recognize membrane-bound HspA1A (Multhoff and Hightower 2011), according to the Multimmune GmbH procedure or with anti-EpCAM FITC-conjugated mAbs for 30 and 10 min, respectively, at 4 °C in the dark. All samples were fixed with BD CellFIX solution and stored at 4 °C until the analysis on a Beckman Coulter (USA) flow cytometer equipped with Cytomics FC 500 MPL System with MXP Software for cell acquisition and data analysis. The neutrophils and OC cells were gated on the forward and side scatter (FSC versus SSC). The expression of membrane-bound HspA1A was measured in viable (PI-negative) and dead (PI-positive) cells. The results were presented as median fluorescence intensity (MFI), which correlates with the expression of the surface molecule. MFI was determined by subtracting the MFI of untreated cells (isotype) from the MFI of positive-stained cells with the appropriate mAbs.

### Detection of released HspA1A

OC cells (1 × 10^5^ in HBSS) were distributed into 96-well plates, and 1 μg/ml of PMA or 1 μM of fMLP was added or not into the wells and cells were then incubated for 60 min at 37 °C in a 5 % CO_2_ atmosphere. The incubation time was adjusted to the time of OC cell–neutrophil interaction during ROS production. PMA and fMLP were added to verify whether these stimulators could affect the OC cells to release HspA1A. Plates were centrifuged (250×*g*, 10 min) and supernatants were collected and stored at −85 °C. In a separate experiment, three cell lines (A2780, SK-OV-3, and OVCAR-3) were cultured for 72 h in 24-well plate (10^6^ cells/well) in the CM. After that, culture supernatants were collected and stored at −85 °C. The quantitative analysis of HspA1A in supernatants was performed by ELISA assay using an appropriate Hsp72 ELISA kit with the sensitivity of 200 pg/ml. The presence of HspA1A in the supernatants was also detected with western blot analysis (described below). Supernatants were obtained from cell lines and OC cells. The culture contained from 1.2 × 10^6^ to 1.8 × 10^6^ cell/ml.

### Western blot analysis

OC cells from tumors (5 × 10^6^ cells in HBSS) were lysed for 30 min on ice in the lysis buffer containing 1 % Triton X-100, 20 mM Tris, 150 mM NaCL, pH 7.4, and supplemented with 1 mM PMSF, 5 mM NaF, 2 mM sodium orthovanadate, 1 mM EGTA, 1 mM EDTA, and 1 % protease inhibitor cocktail. A2780, SK-OV-3, and OVCAR-3 cell lines were cultured for 72 h in 24-well plates (10^6^ cells/well). Then, cells were removed from plates using Trypsin–EDTA solution, centrifuged, suspended in HBSS (5 × 10^6^ cells/ml), and lysed as described above. The protein concentration in the lysates was determined using a DC Protein Assay Kit. The collected supernatants (as described earlier) and lysates were mixed up 1:1 with 2× SDS–polyacrylamide gel electrophoresis (PAGE) sample buffer (125 mM Tris, pH 6.8, 4 % SDS, 2 % β-mercaptoethanol, 20 % glycerol, 0.02 % bromophenol blue) and boiled for 5 min. Samples containing an equal amount of protein were run on 10 % SDS–PAGE minigel. The proteins were transferred to PVDF membranes for 18 h at 4 °C at 35 mA. Membranes were blocked using 0.5 % BSA and 1 % PEG 3500 in 2× TBS–Tween (25 mM Tris, 190 mM NaCL, 10 mM NaF, 0.2 % Tween 20) for 2 h. Then, membranes were incubated with mouse IgG1 anti-inducible form of Hsp72 (SPA810) or with mouse IgG2a anti-β actin monoclonal antibodies for 2 h at room temperature and washed five times in 2× TBS–Tween. Afterward, membranes were incubated with HRP-conjugated goat anti-mouse IgG, IgA, and IgM (1:1,000 in blocking buffer) for 1 h and again washed five times in 2× TBS–Tween. HspA1A and β-actin bands were visualized using the enhanced chemiluminescence (CL) system (Santa Cruz Biotechnology, USA). Actin expression was used as an internal (loading) control. Densitometric analysis of the visualized bands was performed using FluoroChem MultiImage FC Cabinet (Alpha Innotech Corporation) and Alpha Ease FC software 3.1.2. The results were presented as the optical density intensity of the area under each band’s peak (ODI).

### ROS production

The production of ROS molecules by neutrophils was assessed with a luminol-enhanced CL method. Neutrophils (2 × 10^6^ cells/ml in HBSS) were treated with 20 μg/ml of anti-TLR2 and anti-TLR4 sodium azide-free blocking mAbs for 30 min at room temperature or with a 10 μM of specific inhibitor of IRAK1 and IRAK4 kinases (IRAK1/4 inhibitor) for 30 min at 37 °C or remained untreated. Afterward, 50 μl of neutrophils’ suspension (1 × 10^5^ cells/well) and 25 μl of OC cells suspension (1 × 10^5^ cells/well) were distributed into 96-well plates and co-incubated for 30 min (37 °C, 5 % CO_2_). Subsequently, 1 μg/ml of PMA or 1 μM of fMLP to initiate the ROS production and 10 μM of luminol to enhance CL were added to cells.

In the separate experiments, neutrophils after pre-treatment or not with inhibitor IRAK1/4 were distributed into 96-well plates and incubated with exogenous human recombinant HspA1A (hrHspA1A; 5 ng or 10 ng/ml) for 30 min (37 °C, 5 % CO_2_). Next, the production of ROS in neutrophils was induced as described above. In separate experiments, 50 μl of neutrophils’ suspension (1 × 10^5^ cells/well) was pre-incubated with 25 μl OC cell supernatant (obtained as described in “Materials and methods”) for 30 min (37 °C, 5 % CO_2_) and then stimulated with PMA or fMLP (see above).

DMSO was used as a diluent to prepare fMLP and PMA solutions and was checked for a possible stimulating effect on neutrophils. DMSO solution prepared in HBSS, at the final concentrations of 0.1 %, did not affect ROS production by neutrophils over basal levels (in comparison to unstimulated cells in HBSS buffer). The values of RLU total in neutrophils in the presence of the diluent at concentrations used were included in the figures.

The CL reading was recorded for 30 min, with 2-min intervals, on a Fluoroscan Ascent FL fluorometer (Labsystem, Finland). CL intensity was given in relative light units (RLU). Data were expressed as RLU total values, calculated as the area under the curve of CL versus assay time.

### Interaction of hrHspA1A with TLR2 and TLR4

Interaction of hrHspA1A with TLR2 and TLR4 on human neutrophils was determined by FCM. Neutrophils (2 × 10^6^) from healthy individuals were pre-incubated or not with 20 μg/ml of anti-TLR2 and anti-TLR4, sodium azide-free, blocking mAbs for 30 min, and next, cells were incubated with or without hrHspA1A (250 ng/ml, 30 min, 37 °C, 5 % CO_2_). Afterward, neutrophils were stained with anti-human Hsp72 (SPA810) FITC conjugated (30 min at 4 °C in the dark) to detect the presence of receptor-bound HspA1A (Multhoff and Hightower 2011).

In separate experiments, neutrophils were incubated with or without exogenous 250 ng/ml of hrHspA1A or 250 ng/ml of BSA (30 min, 37 °C, 5 % CO_2_). Then, cells were stained with anti-TLR2 PE-conjugated and anti-TLR4 FITC-conjugated mAbs or with 10 μl of appropriate isotype control for 30 min at 4 °C in the dark. All cell samples were fixed and analyzed as described above.

### Statistical analysis

Data are presented as the mean±SD. Statistical analysis of the TLRs expression on neutrophils of cancer patients versus a control group was performed with a nonparametric Mann–Whitney *U* test. The statistical significance of the effect of OC cells and hrHspA1A on neutrophils was verified with Wilcoxon’s signed-rank test. The Statistica 8.0 (StatSoft, Poland) software package was used for data statistical calculations. Statistical significance was defined as *p* ≤ 0.05.

## Results

### Surface expression of TLR2 and TLR4 on neutrophils

**Fig. 1:**
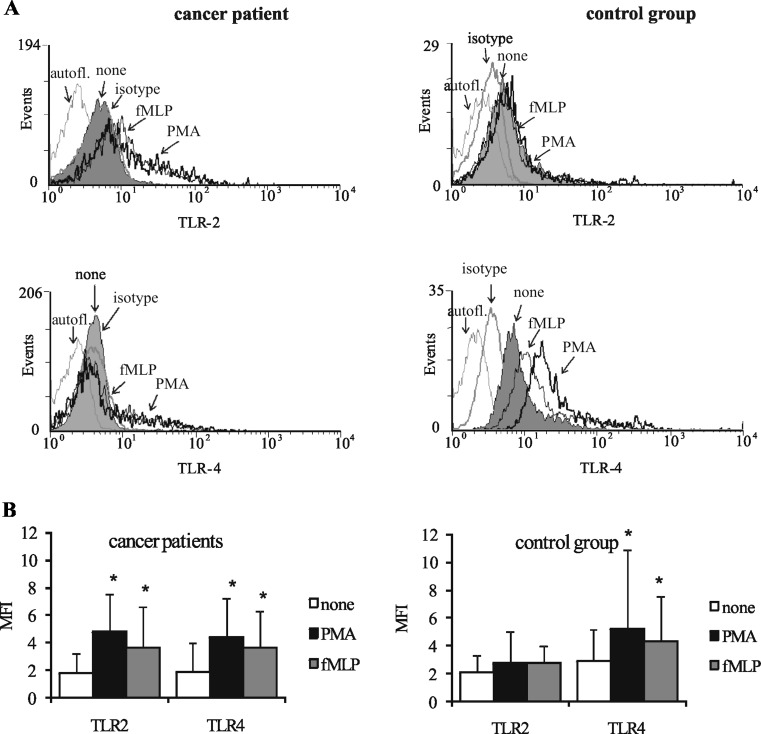
Flow cytometric analysis of TLR2 and TLR4 expression on neutrophils. Neutrophils of cancer patients and control group were incubated with 1 μg/ml of PMA, 1 μM of fMLP, or medium with diluent (0.1 % DMSO) for 30 min prior to staining. **a** Representative histograms show TLRs expression on neutrophils of cancer patient and control group. Isotype control Ab-stained cells are shown as *bold*, *gray line* and untreated cells (none) as gray histograms. *Solid lines* and *arrows* indicate neutrophils treated with PMA or fMLP. Cells in the absence of FITC or PE are marked as an autofluorescence. **b** Graph demonstrates mean values of the median fluorescence intensity (MFI) ± SD (*n* = 15). MFI was determined by subtracting the MFI of isotype control Ab-stained cells from the MFI of positive-stained cells that had been incubated with PMA, fMLP, or medium. *p ≤ 0.05, unstimulated neutrophils versus PMA- or fMLP-stimulated neutrophils, Wilcoxon’s signed-rank test

**Fig. 2:**
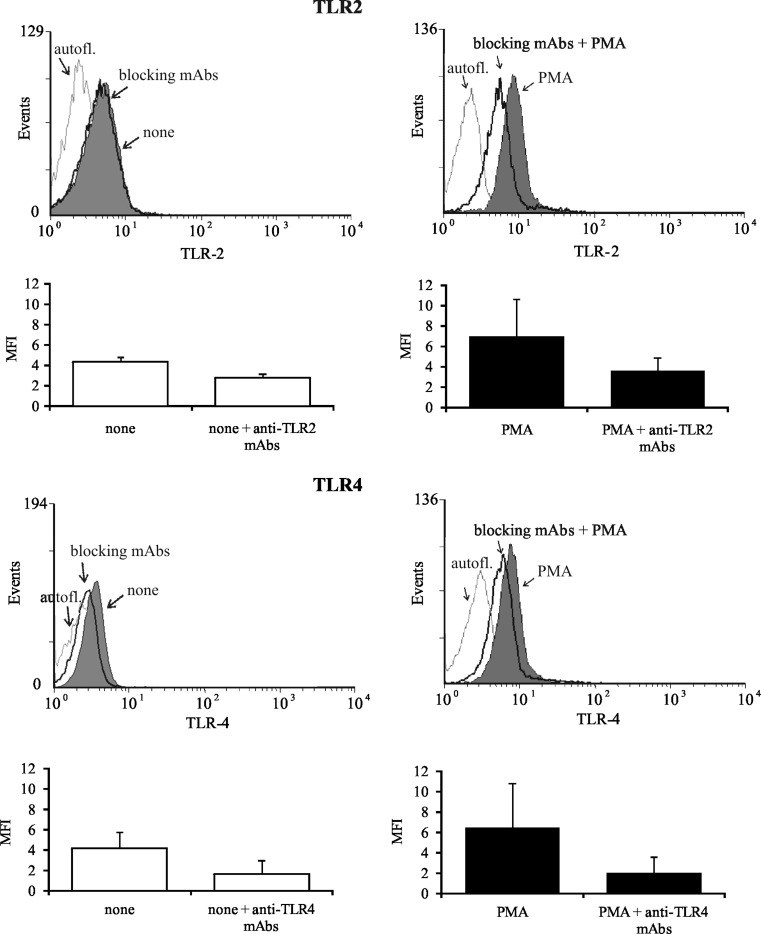
Expression of TLR2 and TLR4 on unstimulated and PMA-stimulated neutrophils in the presence of anti-TLR2 and anti-TLR4 blocking mAbs. Neutrophils of cancer patients were pre-treated with 20 μg/ml of anti-TLR2 and anti-TLR4, sodium azide-free, blocking mAbs for 30 min at room temperature or remained untreated (none). Next, cells were stimulated or not with PMA (1 μg/ml). Representative histograms show TLR2 and TLR4 expression on unstimulated (*left*, *gray-filled histogram*) and PMA-stimulated neutrophils (*right*, *gray-filled*). The *solid black lines* represent unstimulated and PMA-stimulated neutrophils incubated with blocking mAbs. The *solid gray lines* represent the autofluorescence (cells in the absence of FITC or PE). Graph demonstrates mean values of the median fluorescence intensity (MFI) ± SD

We tested whether the effect of OC cells on neutrophils is mediated by TLRs. The expression of TLR2 and TLR4 on the surface of freshly isolated as well as PMA- or fMLP-stimulated neutrophils of cancer patients versus healthy individuals (control group) was assessed using FCM. As shown in Fig. 1, there were no statistical differences between the expression of TLR2 and TLR4 on unstimulated neutrophils of cancer patients and healthy donors. However, we found that exposure of cancer patients’ neutrophils to PMA or fMLP significantly enhanced the expression of both types of TLRs (Fig. 1). The increase in values of TLR2 MFI from 1.7 ± 1.4 to 4.8 ± 2.7 and to 3.6 ± 3.0 and in values of TLR4 MFI from 1.8 ± 2.1 to 4.4 ± 2.8 and to 3.6 ± 2.6 was noted, after stimulation with PMA and fMLP, respectively. In contrast, after the same stimulation of the control group neutrophils, only TLR4 expression was increased and MFI values rose from 2.9 ± 2.2 to 5.7 ± 5.2 and to 4.3 ± 3.2, respectively. Since the expression of TLRs on cancer patients’ neutrophils after incubation with PMA and fMLP was similar, the following experiments were carried out on PMA-stimulated cells only. After pre-incubation of patients’ neutrophils with anti-TLR2 and anti-TLR4 blocking mAbs, the expression of TLR2 and TLR4 on PMA-stimulated neutrophils was reduced, as shown by a decrease of MFI values from 7.0 ± 3.6 to 3.6 ± 1.3 and from 6.5 ± 4.3 to 2.0 ± 1.6, respectively. After pre-incubation of unstimulated neutrophils with these mAbs, the expression of TLR2 and TLR4 also decreased, and MFI values ranged from 4.3 ± 0.5 to 2.5 ± 0.4 and from 4.2 ± 1.6 to 1.6 ± 1.4, respectively (Fig. 2). DMSO as a diluent of fMLP and PMA does not affect the staining of TLRs (results not shown).

### Surface, extracellular, and intracellular location of HspA1A in OC cells

**Fig. 3:**
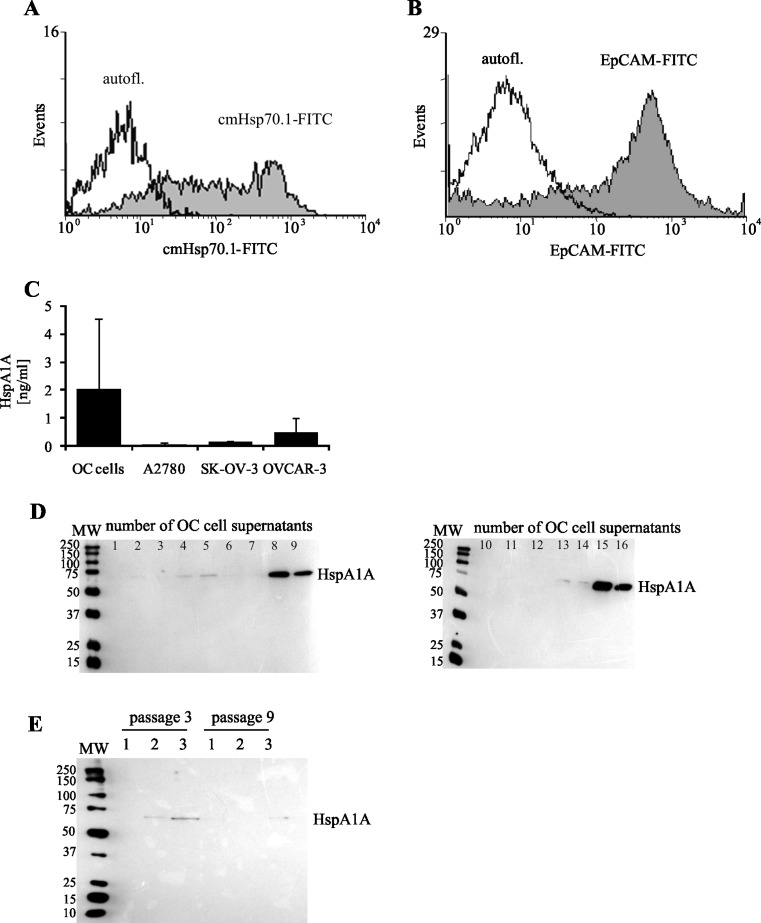
Surface and extracellular expression of HspA1A. The representative histograms of **a** HspA1A and **b** EpCAM expression on OC cells are demonstrated. OC cells were stained with cmHsp70.1 FITC-conjugated mAbs against membrane-bound HspA1A or with EpCAM FITC-conjugated mAbs for 30 and 10 min, respectively, at 4 °C in the dark. The molecules expression was determined using FCM assay. The *gray-filled histograms* represent OC cells exposed membrane-bound HspA1A (**a**) and EpCAM (**b**) molecules. The *solid lines* (*open histograms*) represent the cells in the absence of FITC (autofluorescence). **c** The amounts of HspA1A present in the supernatants from OC cells and from the ovarian cancer cells lines culture measured using ELISA assay. The data are presented as means ng/ml ± SD, for OC cells (*n* = 25) and for cancer cell lines (*n* = 3). Western blot analysis of extracellular HspA1A released **d** from OC cells and **e** from ovarian cancer cell lines. *Lanes*: *1* A2780, *2* SK-OV-3, *3* OVCAR-3. OC supernatants from 16 separate samples and ovarian cancer cell line culture supernatants from two separate passages (as indicated) were analyzed

In this study, we verified the hypothesis whether membrane-bound HspA1A on OC cells or HspA1A released by them is responsible for the activation of neutrophils during OC cell–neutrophil interaction. Flow cytometric analysis showed that nearly 80 % of OC cells expressed membrane-bound HspA1A (MFI value 187 ± 27) and EpCAM molecule (Fig. 3a, b, respectively). Precise analysis also showed that among 63 ± 14 % (47–88 %) of cells expressing the membrane-bound HspA1A, 24 ± 8 % (12–36 %) of cells were PI negative (viable).

The amount of extracellularly released HspA1A from OC cells was estimated in supernatants collected after incubation of OC cells with or without stimuli in vitro as described in “Materials and methods.” OC cells released a small but detectable amount of HspA1A (2.0 ± 2.5 ng/ml) (Fig. 3c). The presence of PMA and fMLP did not influence the amount of HspA1A in OC cells supernatant (2.25 ± 1.25 and 1.98 ± 1.88 ng/ml, respectively). For comparison, A2780, SK-OV-3, and OVCAR-3 cell lines released below 1 ng/ml of HspA1A (0.05 ± 0.07, 0.14 ± 0.02, and 0.48 ± 0.52 ng/ml, respectively) during 72 h of culture (Fig. 3c). Furthermore, we observed that cell lines differed significantly between themselves in the amount of released heat shock protein. The presence of HspA1A in the supernatant from OC cells, as well as in A2780, SK-OV-3, and OVCAR-3 cell line cultures, was also documented by western blot assays (Fig. 3d, e).

It has been reported by others that exosomes secreted from peripheral blood cells, macrophages, dendritic cells, or epithelia cells may provide a secretory pathway that facilitates the active release of specific heat shock proteins (Asea 2007a; Lancaster and Febbraio 2005; Multhoff and Hightower 1996; Vega et al. 2008). To rule out the assumption that HspA1A is also present within exosomes derived from OC cells and the possibility that exosomes may be mediators of HspA1A active release from these cells, the samples of supernatants were treated with a 1 % Triton X-100 solution. This mild detergent solubilizes exosomes, which leads to the release of its internal content. We did not observe any differences between the levels of HspA1A detected in supernatants before and after the treatment of cells with 1 % Triton X-100 solution (1.03 ± 0.3 versus 0.90 ± 0.14 ng/ml, respectively, *n* = 4). This suggests that HspA1A is released from OC cells as a free protein into the extracellular milieu and that the various mechanisms are involved in different cell types to facilitate the release of HspA1A (Asea 2007a). Interestingly, in our preliminary experiments, we did not find any detectable release of HspA1A by neutrophils.

**Fig. 4:**
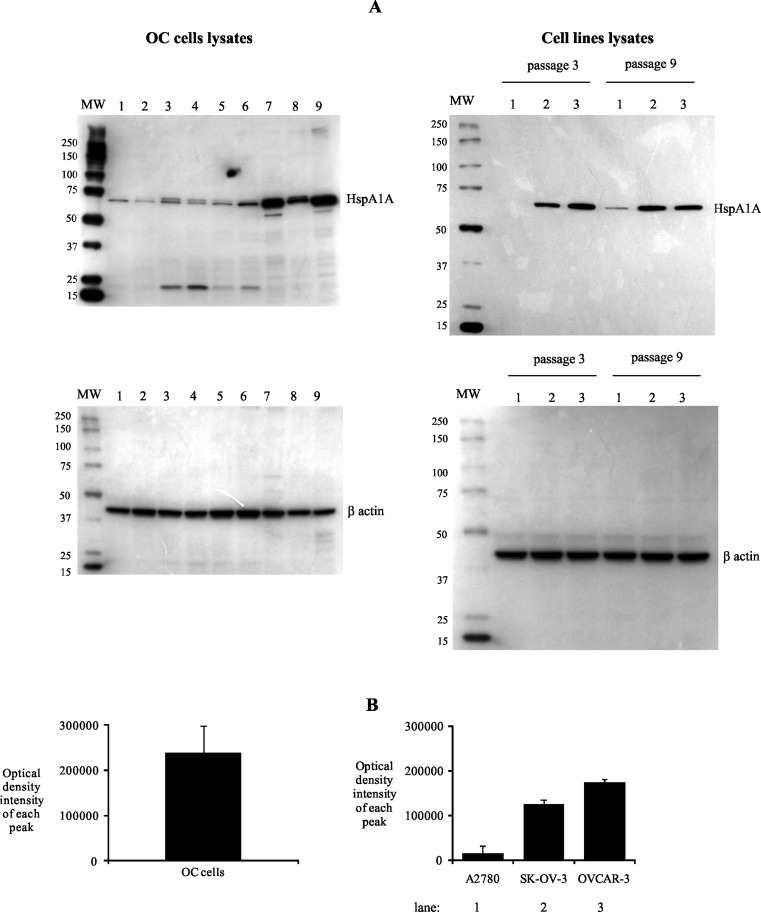
Intracellular expression of HspA1A. OC cells from tumors or ovarian cancer cell lines (cultured for 72 h) were lysed, and the intracellular presence of HspA1A was detected using western blot assay, as described in “Materials and methods.” Representative western blot analysis of HspA1A located (**a**) in OC cells from seven individuals and in ovarian cancer cell lines from three and nine passages. *Lanes*: *1* A2780, *2* SK-OV-3, *3* OVCAR-3. **b** Densitometric analysis of bands. The data are expressed as the mean values of optical density intensity of the area under each band’s peak ± SD, *n* = 25 for OC cells and *n* = 3 for cell lines

The intracellular location of HspA1A was detected in OC cells from examined tumor specimens, as well as in ovarian cancer cell lines (Fig. 4a). However, we observed that OC cells from various tumor samples differed in the amount of intracellular HspA1A and values of ODI ranged from 101,896 to 340,623. Similarly, A2780, SK-OV-3, and OVCAR-3 cell lines demonstrated intracellular HspA1A but its amount in lysates of mentioned cell lines was smaller than in lysates of OC cells. The ODI values of each peak, according to our evaluation, were almost 10-fold lower in comparison to those detected in OC cells and amounted to 14,716, 125,369, and 173,691, respectively (Fig. 4b).

### Production of ROS by neutrophils in the presence of OC cells

**Fig. 5:**
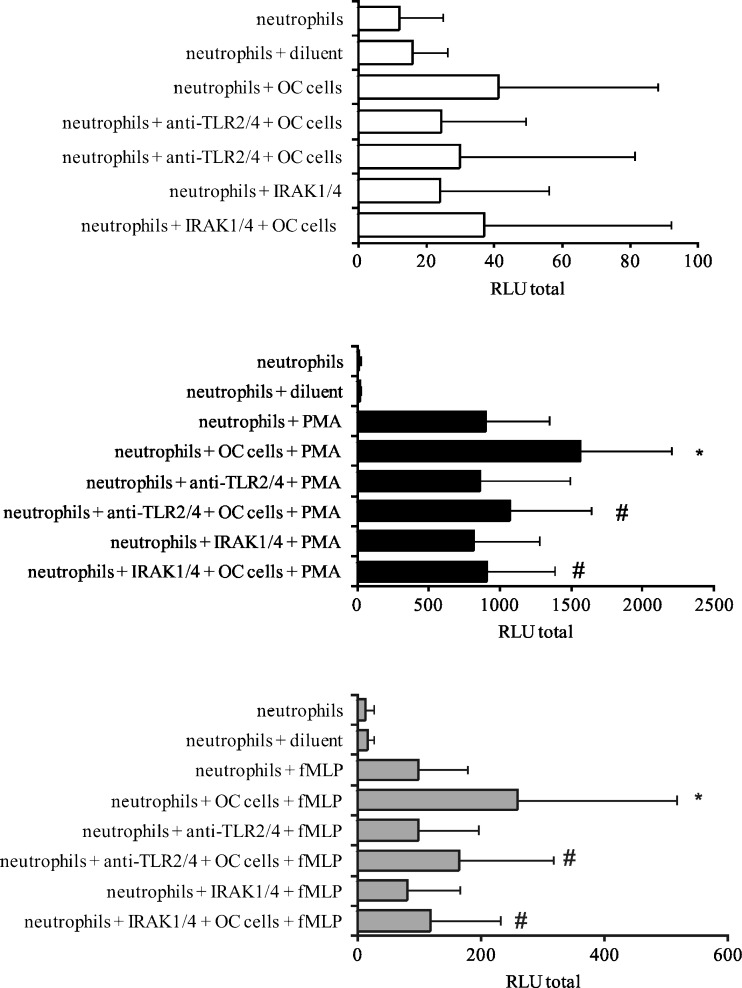
ROS production by neutrophils in the presence of OC cells. Neutrophils were pre-treated with 20 μg/ml of anti-TLR2 or anti-TLR4 mAbs or with IRAK1/4 inhibitor (10 μM) for 30 min or left untreated. After that, neutrophils were co-incubated with OC cells for 30 min and then stimulated with PMA, fMLP, and HBSS with diluent (0.1 % DMSO) or left unstimulated. ROS production was evaluated using the luminol-enhanced CL assay. Data are expressed as mean values of relative light unit total (RLU total) ± SD, *n* = 15. *p ≤ 0.01, neutrophils versus neutrophils + OC cells, Wilcoxon’s signed-rank test. #*p* ≤ 0.01, neutrophils + OC cells versus neutrophils + OC cells + anti-TLR2/4 or IRAK1/4, Wilcoxon’s signed-rank test

Both in our earlier work (Klink et al. 2008) and in our current, we have demonstrated the interaction of OC cells primed neutrophils to enhanced ROS production, in response to subsequent stimulation with PMA and fMLP. In this study, we examined whether such activation of neutrophils by OC cells occurred through the interaction of TLR2 and TLR4 exposed on neutrophils with released and/or expressed HspA1A on OC cells. To assess the participation of TLRs, neutrophils were pre-treated with a saturating concentration of specific anti-TLR2 and anti-TLR4 blocking mAbs (20 μg/ml) for 30 min prior to the addition of OC cells. Pre-incubation with the mentioned mAbs led to the total inhibition of OC cells’ ability to prime neutrophils, as shown by a decrease in the ROS production. As shown in Fig. 5, the values of RLU total decreased from 1,582 ± 633 for neutrophils + OC cells + PMA to 1,073 ± 567 for neutrophils + anti-TLRs mAbs + OC cells + PMA. Similarly, the values of RLU total decreased from 259 ± 257 for neutrophils + OC cells + fMLP to 164 ± 152 for neutrophils anti-TLRs mAbs + OC cells + fMLP. To compare, the values of RLU totals for PMA- and fMLP-stimulated neutrophils were 889 ± 448 and 97 ± 80, respectively.

The mediating role of TLR2 and TLR4 in ROS production by neutrophils, after pre-incubation with OC cells, was also confirmed in the experiments, in which the signaling molecules, such as IRAK1 and IRAK4, that are associated with the activation of TLR2 were inhibited by its specific inhibitor. We found that the neutrophils response to PMA and fMLP stimulation in the presence of OC cells and IRAK1/4 inhibitor (RLU total values were 907 ± 479 and 117 ± 113, respectively) corresponded with their response observed in the absence of OC cells (RLU total values were 898 ± 448 and 97 ± 80, respectively).

**Fig. 6:**
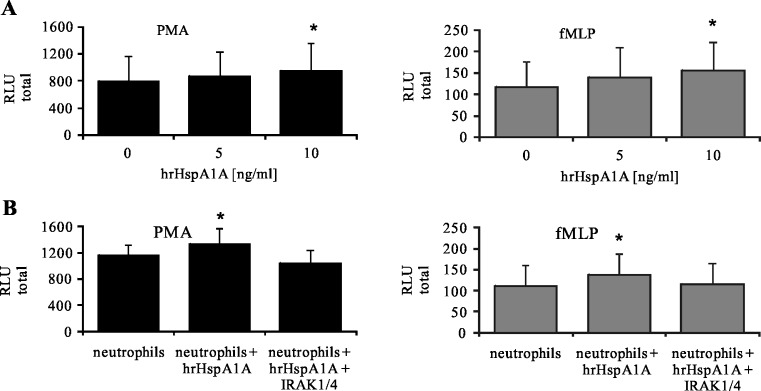
ROS production by neutrophils in the presence of hrHspA1A. **a** Neutrophils were pre-incubated with exogenous HspA1A (hrHspA1A, 5 and 10 ng/ml) for 30 min and then stimulated with PMA or fMLP. **b** Neutrophils were pre-treated or not with IRAK1/4 inhibitor (10 μM) for 30 min, then incubated with hrHspA1A (10 ng/ml) for 30 min, and stimulated with PMA or fMLP. ROS production was assessed using the luminol-enhanced CL assay. Data are expressed as mean values of relative light unit total (RLU total) ± SD, *n* = 6. *p ≤ 0.05, neutrophils versus neutrophils + hrHspA1A, Wilcoxon’s signed-rank test

To prove the hypothesis that HspA1A is involved in the activation of neutrophils, two separate experiments were carried out. In the first experiment, neutrophils were incubated with hrHspA1A and in the second one with supernatant containing HspA1A derived from OC cells. We found that treatment of neutrophils with exogenous hrHspA1A at the concentration of 10 ng/ml caused a significant enhancement of ROS production by neutrophils in response to stimulation with PMA and fMLP (Fig. 6a). To confirm that HspA1A influences neutrophils via TLR2 and TLR4, neutrophils were pre-treated with IRAK1/4 inhibitor prior to incubation with exogenous hrHspA1. We demonstrated in Fig. 6b that such a treatment of cells completely protected neutrophils against the priming effect of hrHspA1A, as shown by a decrease in the RLU total values from 1,326 ± 238 for neutrophils + hrHspA1A + PMA to 1,033 ± 212 for neutrophils + IRAK1/4 + hrHspA1A + PMA. Similarly, we observed a decrease in RLU total values from 137 ± 31 for neutrophils + hrHspA1A + fMLP to 115 ± 19 for neutrophils + IRAK1/4 + hrHspA1A + fMLP. RLU total values for neutrophils + PMA or fMLP were 1,155 ± 169 and 111 ± 18, respectively.

**Fig. 7:**
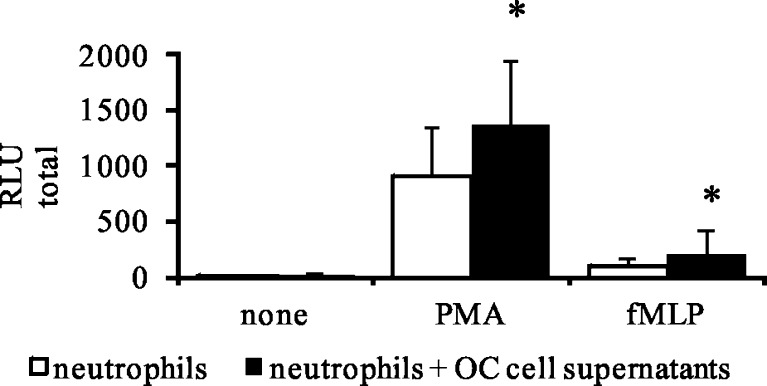
ROS production by neutrophils in the presence of OC cells supernatants. OC cells were incubated in HBSS for 60 min at 37 °C in a 5 % of CO_2_ atmosphere. Then, cells were centrifuged and supernatants were collected. Neutrophils were incubated with OC cells supernatants for 30 min prior to stimulation with PMA or fMLP. ROS production was assessed using the luminol-enhanced CL assay. Data are expressed as mean values of relative light unit total (RLU total) ± SD, *n* = 9. *p ≤ 0.05, neutrophils vs. neutrophils + hrHspA1A, Wilcoxon’s signed-rank test

We also examined whether HspA1A, derived from OC cells, could prime the neutrophils to enhance ROS response to stimuli. We found that the addition of OC supernatants pre-activated neutrophils to enhance ROS production in response to stimuli. The RLU total values enhanced from 899 ± 488 to 1,362 ± 588 for neutrophils treated with PMA and from 97 ± 79 to 201 ± 220 for neutrophils treated with fMLP, as shown in Fig. 7.

### Interaction of HspA1A with the neutrophils TLR2 and TLR4

**Fig. 8:**
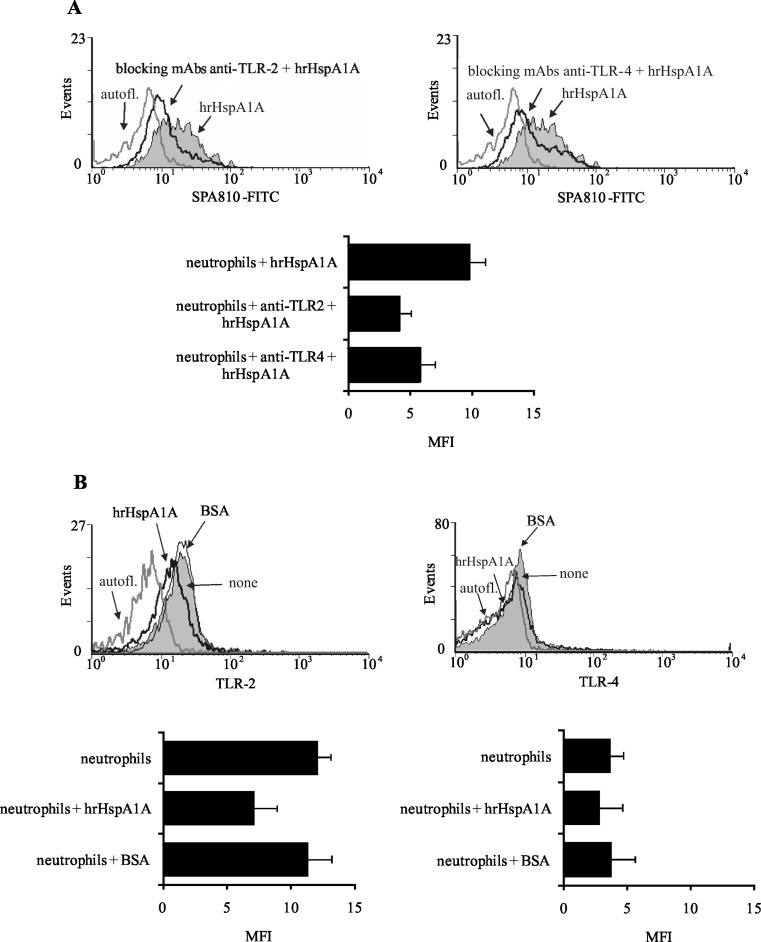
Interaction of hrHspA1A with TLR2 and TLR4 on neutrophils. **a** Neutrophils were pre-treated or not with 20 μg/ml of anti-TLR2 or anti-TLR4 blocking mAbs for 30 min and then incubated with 250 ng/ml of hrHspA1A for 30 min. After that they were stained with anti-HspA1A mAbs (SPA810-FITC). Representative histograms show the receptor-bound HspA1A expression on neutrophils treated with anti-TLR blocking mAbs*. Gray-filled histograms* represent receptor-bound HspA1A. The *solid black lines* represent receptor-bound HspA1A after neutrophils incubation with anti-TLRs mAbs; *gray lines* represent autofluorescence. Graph demonstrates the values of median fluorescence intensity (MFI) ± SD, *n* = 3. **b** Neutrophils were pre-treated with 250 ng/ml of hrHspA1A or with 250 ng/ml of BSA for 30 min and then stained with anti-TLR2 or anti-TLR4 mAbs. Representative histograms show the TLR2 and TLR4 expression on neutrophils. *Gray-filled histograms* represent TLRs expression. The *solid black lines* represent TLR expression after neutrophils incubation with hrHspA1A. The *gray lines* represent autofluorescence and expression of TLRs after neutrophils incubation with BSA as *arrows* indicated. *Graphs* demonstrate the values of MFI ± SD of TLRs on neutrophils, *n* = 3

The interaction of HspA1A with TLR2 and TLR4 has been described by other authors (Asea 2006; Giraldo et al. 2010). Nonetheless, to verify the viability of our studies, the capacity of exogenous HspA1A to bind to TLR 2 and TLR4 on neutrophils was evaluated in two separate control experiments by FCM. Flow cytometric analysis revealed that the ability of hrHspA1A to bind to neutrophils was markedly reduced, after treatment of neutrophils with mAbs blocking TLR2 and TLR4 expression, as shown by the decrease in the value of MFI of HspA1A expression (Fig. 8a). Data presented in Fig. 8b also showed that when neutrophils were incubated with hrHspA1A, the expression of TLR2 and TLR4 was significantly decreased. To test the specificity of the interaction of hrHspA1A with neutrophils’ TLRs, the cells were incubated with BSA. As shown in Fig. 8b, BSA did not influence the expression level of neutrophils’ TLRs.

## Discussion

Our current and previous studies (Klink et al. 2008) demonstrated that OC cells from tumor tissues of patients with advanced ovarian cancer pre-activated autologous blood neutrophils to enhanced ROS production, in response to stimulation with PMA and fMLP. ROS are the important molecules that can enhance cancer progression. They facilitate angiogenesis and metastasis through stimulation of the vascular endothelial growth factor production (Ushio-Fukai and Alexander 2004), as well as an activation of metalloproteinases (De Larco et al. 2004). Therefore, it seems highly probable that the appearance of ROS molecules, as a result of neutrophils interaction with OC cells, may support cancer progression. The mechanism responsible for the activation of neutrophils by cancer cells remains unclear.

The main aim of our research was to recognize the molecular mechanism of ovarian cancer cells action on autologous neutrophils in respect to the interaction of HspA1A with TLR2/4. In this study, we have shown that membrane-bound HspA1A was expressed on nearly 80 % of OC cells isolated from tumor specimens of all tested ovarian cancer patients. Similarly, Kleinjung et al. (2003) demonstrated that almost 60 % of cancer cells isolated from head and neck tumors were HspA1A positive. We and others (Mambula and Calderwood 2006; Gastpar et al. 2005) found that ovarian cancer cells isolated from both tumors, as well as ovarian cancer cell lines A2780, SK-OV-3, and OVCAR-3, released HspA1A outside cells. We demonstrated here that OC cells and cell lines also had intracellularly located heat shock proteins. However, cancer cells isolated from tumors expressed considerably higher levels of HspA1A, in comparison to cancer cell lines. This may be the result of unfavorable conditions that occur in the tumor environment. The environment surrounding cancer cells is deficient in oxygen, nutrients, and glucose but is enriched in pro-inflammatory cytokines and cytotoxic agents that enhance the basal level of HspA1A in tumor cells and which, on the other hand, is essential to the growth and survival of cancer cells (Weber and Kuo 2011; Wilson and Balkwill 2002; Daugaard et al. 2005). It was shown by others that interaction of HspA1A with its specific surface receptors caused the activation of various signal transduction pathways resulting in the stimulation of cells. It was also shown that binding of HspA1A to human monocytes activated NF-κB induced a rapid intracellular Ca^+2^ influx and production of IL-1, IL-6, and tumor necrosis factor-α (TNF-α) (Asea et al. 2002; Asea 2006). Among the group of receptors that can bind HspA1A, TLR2, and TLR4 are known to initiate a signal transduction cascade resulting in the cellular response to this heat shock protein stimulation (Asea et al. 2002).

In this study, we found that the presence not only of OC cells but also HspA1A released from them and exogenous hrHspA1A led to enhanced ROS production in neutrophils in response to further stimulation with PMA and fMLP. These findings suggested the hypothesis that the activation of neutrophils by OC cells may be mediated by HspA1A derived from OC cells. Next, we investigated whether the effect of such an interaction of OC cells with neutrophils was mediated by occupation of TLRs on the neutrophils’ surface.

Toll-like receptors are important molecules involved in the initiation of neutrophil activation. Numerous studies indicate that the activation of TLRs enhances the CD11b molecule expression, induces the L-selectin shedding, and stimulates the production of ROS and release of chemoattractants, e.g., IL-8 (Kurt-Jones et al. 2002; Sabroe et al. 2005; Hayashi et al. 2003). It has also been found that the pre-treatment of neutrophils with TLRs agonists resulted in a marked increase in ROS production in response to subsequent activation with fMLP (Sabroe et al. 2003). On the other hand, various stimuli including IL-1β, TNF-α, granulocyte–macrophage colony-stimulating factor, and interferon-γ have been shown to enhance the expression of TLRs on the surface of neutrophils (Kurt-Jones et al. 2002; O’Mahony et al. 2008). In agreement with other reports (Kurt-Jones et al. 2002; O’Mahony et al. 2008), we demonstrated very low constitutive expression of both TLR2 and TLR4 on the surface of neutrophils isolated from the blood of cancer patients, as well as of the control group. We found that fMLP and PMA significantly increased the expression of both TLR2 and TLR4 on ovarian cancer patients’ neutrophils. In contrast to the neutrophils of cancer patients, PMA and fMLP caused an increase in TLR4 but not in TLR2 expression levels on the neutrophils of the control group. This difference is probably caused by the activation status of the cancer patients’ neutrophils. It has been shown that circulating neutrophils of advanced cancer patients are primed in vivo, and their response to stimuli in vitro differs significantly from that of healthy donors’ neutrophils (production of ROS, cytokines, expression of adhesion molecule) (Klink et al. 2008; Jablonska et al. 2005; Goddard et al. 2005).

To confirm that TLR2 and TLR4 are involved in the stimulation of ROS production by OC cells, we studied the signal transduction pathway that can be initiated by these receptors. We noted that the presence of specific anti-TLR2 and anti-TLR4 blocking antibodies, as well as of a specific inhibitor of IRAK1 and IRAK4 signaling molecules, prevented the enhancement of ROS production by OC cells and/or hrHspA1A. This finding suggests that the effect of exogenous hrHspA1A and OC cells (via released or/and membrane-bound HspA1A) on neutrophils is connected with TLRs.

Moreover, by using flow cytometry, we demonstrated that HspA1A bound to TLR2 and TLR4 was expressed on neutrophils. Our data are in agreement with several reports showing that extracellular HspA1A serves as a ligand for TLRs (Asea 2008; Giraldo et al. 2010; Ortega et al. 2009). Others have also documented the ability of extracellular HspA1A to stimulate neutrophil phagocytic function via TLR2 (Giraldo et al. 2010), as well as to induce IL-8 and TNF-α production via TLR4 (Wheeler et al. 2009) and to induce production of pro-inflammatory cytokines via TLR2 and TLR4 (Asea et al. 2002).

In summary, we have described that direct contact of cancer cells isolated from tumors with neutrophils intensified their biological functions (Klink et al. 2008). Currently, our study demonstrates for the first time that activation of ovarian cancer patients’ neutrophils by ovarian cancer cells is dependent on the interaction of HspA1A originating from ovarian cancer cells, with TLR2 and TLR4 expressed on the surface of neutrophils. Our data may have a practical implication for targeted anticancer therapies based, among other factors, on the inhibition of HspA1A expression in the cancer cells. The prevention of the pro-inflammatory activity of neutrophils, enhanced by OC cells (via expressed HspA1A), may protect against tumor progression and metastases.
